# Rare-earth free self-luminescent Ca_2_KZn_2_(VO_4_)_3_ phosphors for intense white light-emitting diodes

**DOI:** 10.1038/srep42348

**Published:** 2017-02-09

**Authors:** L. Krishna Bharat, Soo-Kun Jeon, Kurugundla Gopi Krishna, Jae Su Yu

**Affiliations:** 1 Department of Electronic Engineering, Institute for Wearable Convergence Electronics, Kyung Hee University, Yongin-si, Gyeonggi-do 446-701, Republic of Korea; 2 Semicon Light Co., Ltd., 49 Wongomae-ro 2beon-gil, Giheung-gu, Yongin-si, Gyeonggi-do 446-901, Republic of Korea

## Abstract

The commercially available white-light-emitting diodes (WLEDs) are made with a combination of blue LEDs and yellow phosphors. These types of WLEDs lack certain properties which make them meagerly applicable for general illumination and flat panel displays. The solution for such problem is to use near-ultraviolet (NUV) chips as an excitation source because of their high excitation efficiency and good spectral distribution. Therefore, there is an active search for new phosphor materials which can be effectively excited within the NUV wavelength range (350–420 nm). In this work, novel rare-earth free self-luminescent Ca_2_KZn_2_(VO_4_)_3_ phosphors were synthesized by a citrate assisted sol-gel method at low calcination temperatures. Optical properties, internal quantum efficiency and thermal stability as well as morphology and crystal structure of Ca_2_KZn_2_(VO_4_)_3_ phosphors for their application to NUV-based WLEDs were studied. The crystal structure and phase formation were confirmed with XRD patterns and Rietveld refinement. The optical properties of these phosphor materials which can change the NUV excitation into visible yellow-green emissions were studied. The synthesized phosphors were then coated onto the surface of a NUV chip along with a blue phosphor (LiCaPO_4_:Eu^2+^) to get brighter WLEDs with a color rendering index of 94.8 and a correlated color temperature of 8549 K.

A light-emitting diode (LED) is in the limelight since its invention by Nick Holonyak Jr. of General Electric Company and its application in solid-state lighting industry has gained a prominent interest. In the contemporary situation, white LEDs (WLEDs) have drawn great attention due to their wide range of applications and salient features like high efficiency, compact size, eco-friendly feature, high thermal stability and long operational lifetime^1^,^2^. The commercially available WLEDs are prepared by coating a yellow-emitting Y_3_Al_5_O_12_: Ce^3+^ (YAG: Ce^3+^) phosphor on blue-emitting GaN LEDs^3^,^4^. Such WLEDs have several drawbacks including poor color reproducibility, low color-rendering index (CRI) value and less thermal stability at high temperature. For the sake of its usage in general display applications, there is a need of high CRI and appropriate correlated color temperature (CCT). Therefore, the WLEDs visualized with near-ultraviolet (NUV) LED chips have been turned into a topic of research interest because their excitation efficiency is almost similar to that of fluorescent light bulbs and less than that of blue LED chips^5^,^6^. For blue LED chips, the electroluminescence (EL) intensity considerably increases in the long wavelength band and saturates at high driving current, and the CCT value readily changes. There is also a non-uniformity in the spectral distribution due to low CRI values. On the other hand, NUV LED chips cannot be used to make YAG-based WLEDs due to weak light absorption in the NUV or deep blue spectral region. To overcome this, the search for a novel phosphor which can be excited in the wavelength range of 350–420 nm is building up.

Broadly speaking, rare-earth materials are widely used in preparing host materials and as luminescent centers in many phosphors to generate multi-color lights^7^,^8^,^9^. There are several reports found on rare-earth doped single host materials for WLEDs^10^,^11^,^12^,^13^,^14^,^15^. Since all the rare-earth materials have similar chemical properties, they require high-cost separation, refinement and purification techniques which make them mostly expensive^16^. Due to this, the design of rare-earth free materials is majorly needed. The rare-earth free phosphors can be understood with three main strategies: (i) use of transition metals for luminescent centers^17^,^18^,^19^,^20^,^21^,^22^, (ii) use of defects such as oxygen vacancies^23^,^24^,^25^,^26^ and (iii) use of materials such as tungstates and vanadates^27^,^28^,^29^,^30^.

Generally, vanadate compounds have a wide range of applications in the fields of catalysis, electrochemistry, optical lasers and biology. These materials are also intensively studied as luminescent materials after revealing the optical properties of yttrium vanadate^31^. Subsequently, many vanadate phosphors were developed and studied for their self-luminescence and the effect of rare-earth doping was also investigated^32^,^33^,^34^,^35^. Vanadate materials show visible emissions with UV excitation due to the presence of (VO_4_)^3−^ clusters. In the vanadate cluster, the central metal ion is surrounded by four oxygen ions in tetrahedral symmetry (*T*_*d*_). In some of the vanadate compounds, an unusual luminescent property was observed, i.e., a broad and intense charge transfer (CT) band due to the charge transfer from O^2−^ ligand anion to V^5+^ in the NUV range, thereby yielding a broad emission band over the entire visible range (400–700 nm)^36^,^37^. Furthermore, these materials have considerable advantages over conventional phosphor materials, including low cost and energy efficiency (due to low calcination temperature) as well as broadband emission to obtain high CRI^38^,^39^.

In this article, we report self-luminescent Ca_2_KZn_2_(VO_4_)_3_ (called CKZV, hereafter) vanadate phosphors which provide yellow-green emissions under NUV excitation. Previously, some of the authors reported the synthesis and luminescent properties Ca_2_KMg_2_(V_1x_P_x_)_3_O_12_, Ca_2_KMg_2_V_3_O_12_, Ca_2_NaMg_2_V_3_O_12_ co-doped with Dy^3+^ and Sm^3+^, Ca_2_NaMg_2_V_3_O_12_ doped with Eu^3+ ^31,35,40,41. To the best of our knowledge, there were no reports found on CKZV phosphors. These phosphors can be suitable candidates for making NUV-based WLEDs when combined with a blue phosphor. The phosphor exhibits an internal quantum efficiency which is 1.25 times higher than that of the isostructural compound reported^33^, which makes it suitable for solid-state lighting applications. The CKZV phosphors were synthesized by a citrate assisted sol-gel method and their morphological properties were studied. Rietveld refinement was performed on X-ray diffraction (XRD) patterns to study the structural properties and the self-luminescent properties were analyzed through photoluminescence (PL) excitation (PLE) and PL emission spectra. To obtain brighter white light, the CKZV phosphor was mixed with an approximate amount of a blue LiCaPO_4_: Eu^2+^ phosphor prepared via a solid-state technique to fabricate NUV-based WLEDs. The prepared WLED shows specific optical properties and high CRI (94.8) value which are required for its application in flat panel displays and general illumination.

## Results and Discussion

**Figure 1:**
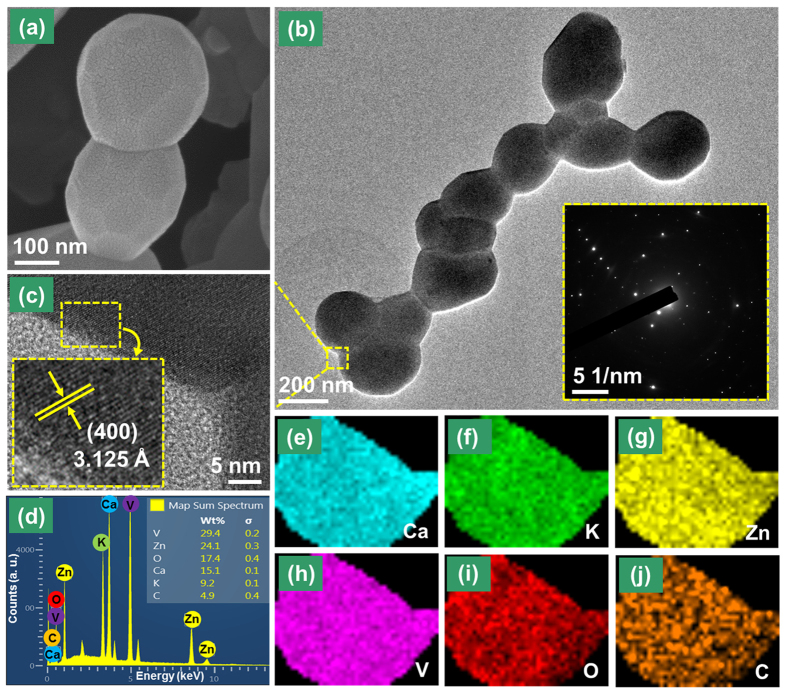
**(a)** FE-SEM image, **(b)** TEM image (inset shows the SAED pattern), **(c)** HR-TEM image (inset shows the magnified HR-TEM image with d-spacing corresponding to the principal plane), **(d)** EDX pattern (inset shows the elemental wt% distribution) and **(e–j)** elemental mappings for the CKZV phosphor.

Figure 1 shows the morphological properties from FE-SEM and TEM analyses for the CKZV sample prepared by a citrate assisted sol-gel method. The formed particles were found to be almost spherical in shape (Fig. 1(a)), which is a useful property required to apply the material for WLEDs. The low magnification SEM image to show the size distribution of the particles is shown in Fig. S1 (Supporting Information). The TEM image (Fig. 1(b)) displays that the synthesized CKZV particles were in the range of few hundred nanometers. The inset of Fig. 1b displays the selective area electron diffraction (SAED) image showing a dot pattern from which it can be said that the particles are single crystalline in nature^42^. The HR-TEM image was taken to further confirm the single crystalline nature of the sample as shown in Fig. 1(c). The inset of Fig. 1(c) shows clear lattice fringes and the calculated d-spacing was found to be ~3.125 Å which matches well with the (400) principal plane. The elements present in the CKZV phosphor and the compositions were confirmed by the energy dispersive X-ray (EDX) spectrum and elemental mapping scan for a small area. The EDX spectrum (Fig. 1(d)) revealed all the elements present in the sample including a small peak at the lower energy, which is related to carbon. The presence of this peak is caused by the carbon tape used in sample preparation. The elemental mappings of all elements present in the CKZV sample are shown in Fig. 1(e–j), which impart and confirm the uniform distribution of elements in the prepared sample.

**Figure 2:**
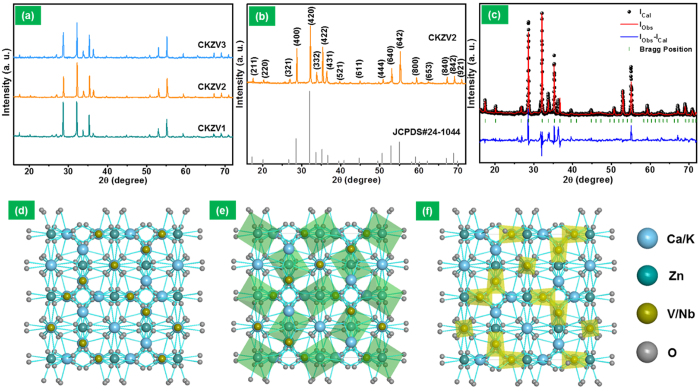
**(a)** XRD patterns of the CKZV phosphors prepared at different calcination temperatures, **(b)** CKZV2 phosphor along with JCPDS card value, **(c)** Rietveld refinement for the CKZV2 phosphor and **(d–f)** crystal structures drawn using the diamond software.

Figure 2 shows the XRD patterns, Rietveld refinement results and crystal structure of the CKZV phosphors. The XRD patterns of the samples calcinated at different temperatures of 600, 650 and 700 °C (called CKZV1, CKZV2 and CKZV3, respectively, hereafter) are shown in Fig. 2(a). The three patterns exhibited all the major and minor peaks related to the CKZV garnet structure, implying the formation of the phase. Figure 2(b) shows the selective XRD pattern of the CKZV2 sample along with the JCPDS card (#24-1044) values. The XRD pattern is well consistent with the standard values and the structure belongs to the space group of Ia-3d (230). The self-luminescent vanadate garnet phase of the compound was further confirmed by Rietveld refinement using the FullProf software. For Rietveld refinement, there is a need of initial structural model being close to the current crystal structure. Herein, Ca_2_NaZn_2_V_3_O_12_ was considered to estimate the structure model because of its isostructural nature to CKZV. The crystallographic and atomic parameters of the unit cell are refined using the Rietveld refinement technique. The final values of the refinement are R_p_ = 5.0, R_wp_ = 6.7 and χ^2^ = 3.72 for the CKZV2 sample. The Rietveld refinement results are shown in Fig. 2(c) and their crystallographic and atomic position data are summarized in Table 1. The Rietveld refinement was also carried out for the CKZV1 and CKZV3 samples and their crystallographic and atomic data were compared in Table S1 (Supporting Information). Employing a Diamond software, an ideal unit cell was imitated utilizing the obtained atomic coordinates, as shown in Fig. 2(d). The perspective view of the unit cell structure of CKZV along the b-axis direction is shown in Fig. 2(d). Here, the Ca and K atoms have ionic radii of 1 and 1.38 Å, respectively, but the difference is close enough to replace each other in the crystallographic site. These atoms occupy the same site with different occupancies of 2/3 and 1/3, respectively. The Ca^2+^ and K^+^ ions form eight-fold dodecahedra (Ca, K)O_8_, the Zn^2+^ ions form a six-fold octahedron (ZnO_6_) and the V^5+^ ions form a four-fold tetrahedra (VO_4_)^32^,^43^. The crystal structure with all the ZnO_6_ octahedra in the unit cell is shown in Fig. 2(e), and the structure with all VO_4_ tetrahedra is similarly shown in Fig. 2(f). In particular, these results revealed that the crystallographic parameters and atomic position of each ion in CKZV are altered when compared to the isostructural Ca_2_NaZn_2_V_3_O_12_ because of the smaller ionic radius of the Na^+^ (1.02 Å) than the K^+^ (1.38 Å).

**Figure 3:**
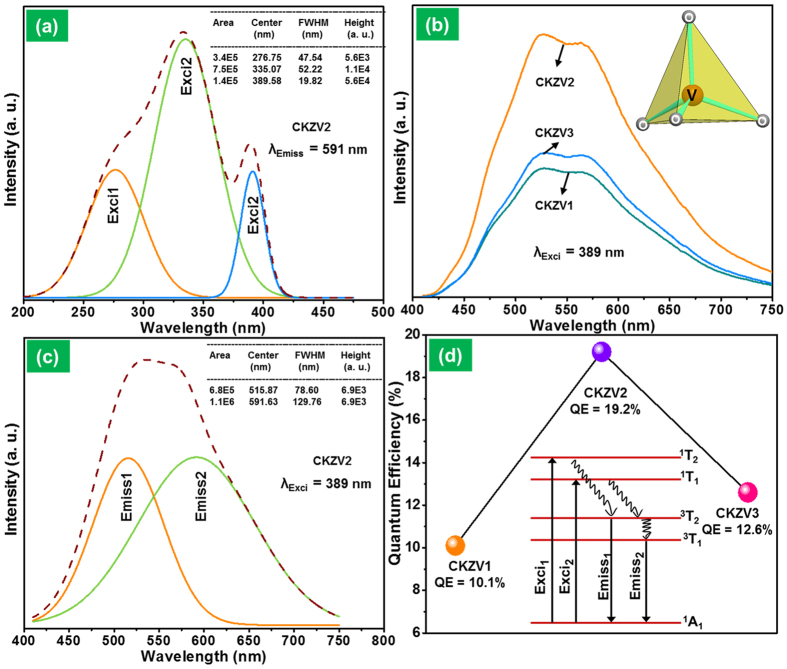
**(a)** PLE spectrum of the CKZV2 phosphor with the Gaussian fitting results, **(b)** PL spectra of the samples prepared at different calcination temperatures, **(c)** PL spectrum with the Gaussian fitting results and **(d)** internal quantum efficiency of the phosphors prepared at different calcination temperatures (inset shows the energy level scheme of the phosphor).

The PLE spectrum for the prepared sample is shown in Fig. 3(a) and it is deconvoled with a Gaussian fitting. The spectrum exhibited three absorption bands centered at 276, 335, and 389 nm, respectively. Usually, the absorption band in vanadate compounds depends on the nature of the host lattice and the concentration of (VO_4_)^3−^ groups^32^. The absorption band for the CKZV2 sample was found to be in the range of 200 to 400 nm which was similar to the previous reports^33^,^44^. The deconvoled absorption band (Exci_1_) at a lower wavelength centered at 276 nm corresponds to the ^1^A_1_ → ^1^T_2_ electronic transition. The intense absorption band (Exci_2_) centered at 335 nm along with the small shoulder band centered at 389 nm corresponds to the ^1^A_1_ → ^1^T_1_ electronic transition. Here, we consider the 389 nm as the excitation wavelength of the phosphor because the UV LED made with this wavelength gives the controlled CRI and a wide range of colors, and it is less harmful to human eyes when compared to deep UV light. Figure 3(b) shows the PL emission spectra of the CKZV samples prepared at different calcination temperatures of 600, 650 and 700 °C when excited at a wavelength of 389 nm. The emission spectra revealed a broadband ranging from 400 to 750 nm and the emission intensity increased with an increase in calcination temperature up to 650 °C and then decreased at 700 °C. By taking this into consideration, the CKZV2 was chosen as the optimized sample. The inset of Fig. 3(b) shows the VO_4_ tetrahedron which is responsible for the broadband emission in the visible region. The broadband emission from the vanadate phosphor consists of two broad peaks related to the ^3^T_1_ → ^1^A_1_ and ^3^T_2_ → ^1^A_1_ transitions of (VO_4_)^3−^ group. This broadband emission is due to the charge transfer (CT) from the oxygen *2p* orbital to the vacant vanadate (V^5+^) ions *3d* orbital. The two broad peaks are overlapped with each other and cannot be seen with naked eye due to the small energy difference between the ^3^T_1_ and ^3^T_2_ excited states (~0.06 eV). Consequently, the broad emission band is deconvoled into two bands by Gaussian fitting, as can be seen in Fig. 3c. The narrow band (Emiss_1_) at the lower wavelength centered at 516 nm corresponds to the electronic transition of ^3^T_2_ → ^1^A_1_, and a slightly broader band (Emiss_2_) at the longer wavelength region is attributed to the ^3^T_1_ → ^1^A_1_ electronic transition of (VO_4_)^3−^ group and the band maximum is observed at 591 nm. The two emission bands were found to be nearly of the same intensity and the relative full width at half maximum values were found to be 79 and 130 nm for Emiss_1_ and Emiss_2_ bands, respectively. The inset of Fig. 3(d) shows the schematic energy level diagram portraying the excitation and emission process. The ^1^A_1_ ground state is from the completely filled *t*_*1*_ molecular orbital and the four excited states, namely, ^1^T_1_, ^1^T_2_, ^3^T_1_ and ^3^T_2_ are from the first excited configuration (*t*_*1*_^*5*^*2e*)^45^,^46^. The internal quantum efficiency was measured for all the samples and the results are shown in Fig. 3(d). The CKZV2 sample has an efficiency of 19.2% which is higher than that of the CKZV1 and CKZV3 samples. The internal quantum efficiency of the CKZV2 phosphor was also compared with some other vanadate phosphors as shown in Table S2 (Supporting Information).

**Figure 4:**
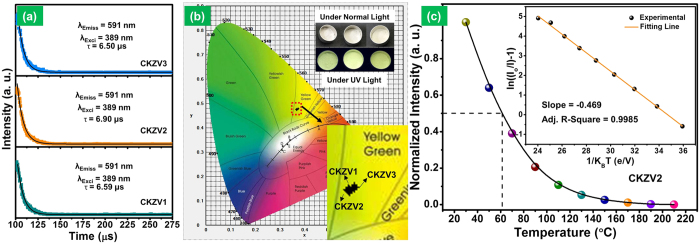
**(a)** Decay curves and **(b)** CIE graph for the samples prepared at different calcination temperatures (lower inset shows the magnified picture of CIE diagram and the upper inset shows the photographs of powder colors under normal and UV light), and **(c)** temperature-dependent emission properties of the CKZV2 phosphor (inset shows the plot of 1/K_B_T vs. ln((Io/I)−1)).

The decay times of the CKZV1, CKZV2 and CKZV3 samples were measured with an excitation wavelength of 389 nm and an emission wavelength of 591 nm and the corresponding curves are shown in Fig. 4(a). The decay curves were well fitted to single exponential functions, and the decay time values were estimated to be 6.59, 6.90 and 6.50 μs for the CKZV1, CKZV2 and CKZV3 samples, respectively. The short decay time of phosphor material avoids saturation at high excitation flux^47^,^48^. So, the short decay time of CKZV samples makes them more suitable as materials for WLEDs. The powders showed a yellow-green emission under UV light while there was no emission under normal light. This emission color was confirmed by calculating the Commission International de I’eclairage (CIE) coordinates for the three samples, which are present in the yellow-green region as shown in Fig. 4(b). The emission color from the powders under UV light irradiation is also shown in the inset of Fig. 4(b), indicating a yellow-green emission from all the samples. Generally, the temperature-dependent emission property was measured to estimate the thermal stability of the prepared phosphor material and it has great influence on the CRI and light output. The temperature-dependent emission spectra (Fig. 4(c)) of the CKZV2 sample were taken in the wavelength range of 400 to 750 nm at temperatures in the range of 30–210 °C (step of 20 °C) and its corresponding activation energy was calculated. The emission spectra do not show any obvious shift and it is beneficial to get color stability at high temperatures^33^. The emission intensity decreased apparently with the increase of temperature due to the non-radiative phonon relaxation from the populated higher energy levels, which causes a quenching in the emission intensity^33^,^37^. The thermal quenching temperature (T_0.5_), specified as the temperature at which the emission intensity becomes half of its original value, was more or less equal to 62 °C. The activation energy (E_a_) for thermal quenching was also calculated from the plot drawn between 1/K_B_T and ln ((I_o_/I)−1), as shown in the inset of Fig. 4(c). The obtained points were fitted linearly and the E_a_ value was calculated from the slope of the fitted line which was found to be ~0.469 eV.

**Figure 5:**
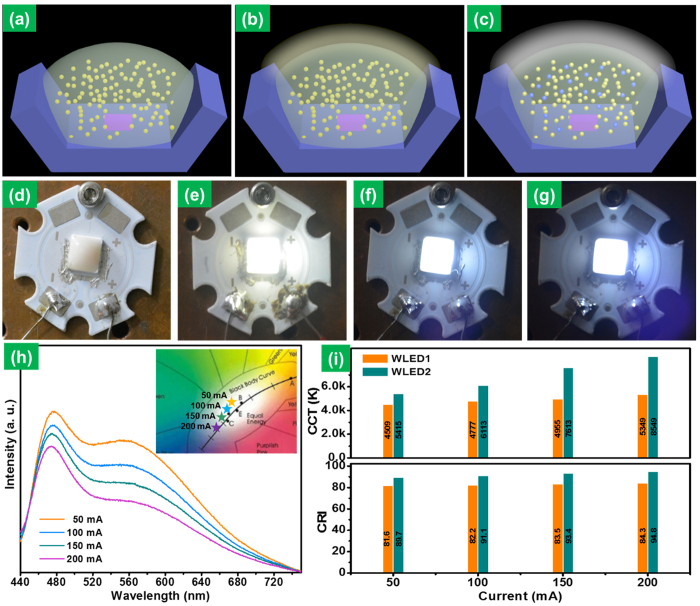
Schematic diagrams showing the WLED with CKZV phosphor (**a**) before applying the current, **(b)** after applying the current and **(c)** WLED with the CKZV and blue phosphors showing the white-light emission under applied forward-bias current. **(d)** Fully packaged WLED. **(e)** WLED1 operated at 50 mA of forward bias current, **(f,g)** WLED2 operated at 50 mA and 200 mA of forward bias current, **(h)** EL spectra of the WLED2 at different operating currents (inset shows CIE diagram for WLED2 at different operating currents). **(i)** CRI and CCT values of WLEDs prepared with and without blue phosphor at different operating currents.

The practical applicability of the prepared phosphor material was tested by fabricating a WLED device. Figure 5(a–c) shows the schematic diagrams for WLED packaging. Here, the WLEDs were prepared by combining the NUV LED chips with the optimized CKZV (i.e., CKZV2) phosphor. The LED fabrication was detailed in the experimental section and the schematic representation was shown in Fig. S2 (Supporting Information). The final samples were then mounted onto MCPCB and copper heat sink to evaluate the cooling effect on the WLEDs. The fully packaged WLED is shown in Fig. 5(d). Two WLED samples were fabricated: WLED1 is a combination of a NUV chip and CKZV2 phosphor and similarly, WLED2 is a combination of a NUV chip with CKZV2 and blue phosphors (LiCaPO_4_: Eu^2+^, lab made phosphor). The optimized weight ratio of CKZV2 and blue phosphors was 0.18 g:0.03 g. The LiCaPO_4_: Eu^2+^ was chosen as a blue phosphor because of its wide range of excitation wavelengths and it also fits the excitation wavelength of the NUV chip and CKZV2 phosphor. The WLED2 gives efficient white-light emission due to the combination of the CKZV2 and blue phosphors. When a forward bias current of 50 mA was applied, a bright yellow-green emission (Fig. 5(e)) and a cool white-light emission (Fig. 5(f)) were observed from the WLED1 and WLED2, respectively. The blue emission intensity in the WLED2 sample increased as the forward bias current increased, which is a useful property to obtain glare-free white-light emission, as can be observed from Fig. 5(g) (WLED operated at 200 mA of forward bias current). The electroluminescence (EL) spectra of the WLED2 at different forward bias currents are shown in Fig. 5(h). The CIE values shifted from white to bluish white with the increase of applied forward bias current as shown in the inset of Fig. 5(h). The CRI and CCT values measured for the WLED1 and WLED2 samples at different operating currents are shown in Fig. 5(i). The CRI value of the WLED2 for an operating current of 50 mA is 89.7 and the CCT value is 5415 K which is higher when compared to the WLED1. The CRI value increased and reached to 94.8 for a forward bias current of 200 mA with a CCT value of 8549 K. When LED operates at a forward bias current, heat is typically generated and this causes a little shift in the NUV LED emission peak which in turn leads to the decrease in the emission intensity at the longer wavelength. To personify this effect, two WLEDs were fabricated with a combination of the CKZV2 and blue phosphors and were placed on heat sinks (metal core plastic chipboard (MCPCB) or copper heat sink). When placed on MCPCB, the sample showed little shift in the NUV LED emission peak with increasing the operating current, which results in the decrease of emission intensity at the longer wavelength. This effect was further reduced by placing it on the copper heat sink with high thermal conductivity because the shift in UV peak is less and the intensity is also comparatively enhanced when compared to the sample placed on the MCPCB. The results were presented in the Supporting Information (Figs S3 and S4).

## Conclusion

In summary, we have successfully synthesized the CKZV yellow-green emitting phosphors which are potential self-luminescent rare-earth free candidates for NUV-based WLEDs. The formed particles were almost spherical and single crystalline in nature, which was confirmed by TEM image, SAED pattern and HR-TEM image. The Rietveld refinement was performed for XRD patterns to confirm the cubic garnet phase of CKZV phosphors. The internal quantum efficiency of the phosphors was improved with the calcination temperature. The CKZV phosphors had the excitation wavelength in the range of NUV LED chip, which is suitable for changing the NUV emission to visible yellow-green emission. The WLED made with only CKZV phosphor showed a yellow-green emission with a CRI value of 84.3 and CCT of 5349 K. The CKZV phosphor, when combined with a broadband blue-emitting LiCaPO_4_: Eu^2+^ phosphor, exhibited the increased CRI value of 94.8 and a brighter white light was observed from the WLED. From these results, the CKZV phosphors can be expected as a promising candidate for applications in solid-state lighting.

## Experimental Section

### Materials

Calcium nitrate tetrahydrate (Ca(NO_3_)_2_·4H_2_O), potassium nitrate (KNO_3_), zinc nitrate hexahydrate (Zn(NO_3_)_2_·6H_2_O), ammonium metavanadate (NH_4_VO_3_) and citric acid (HCO(COOH)(CH_2_COOH)_2_) were employed as source materials. All the chemicals which are of analytical grade were purchased from Sigma-Aldrich Co. and used as received without any further purification. The de-ionized water (aqua dest) used in this experimental process was acquired from a Milli-Q synthesis system (resistivity of 18.2 MΩ-cm).

### Preparation of CKZV Phosphors

The CKZV phosphor powders were prepared by a citrate assisted sol-gel method. Stoichiometric amounts of metal nitrates and ammonium metavanadate were added to aqua dest (200 ml) and stirred for 20 min to form a homogenous solution. Later, citric acid was added as a complexing agent in 1:2 ratio (metal to citric acid) and was capped and stirred for 30 min more. The final solution while capped was heated from room temperature to 80 °C on a hotplate, and after 1 h of continuous stirring and heating, the cap of the beaker was removed and the solution was made to evaporate. The final gel was collected and dried in the oven at 120 °C for 10 h and then calcinated in the muffle furnace. The gel was calcined at different temperatures of 600, 650 and 700 °C for 5 h with an intermediate heating at 400 °C for 3 h to obtain a bright yellow powder and it was further characterized. The obtained powders were then washed with aqua dest to remove the impurities.

### Characterization of CKZV Phosphors

The as-prepared powders were characterized by using a scanning electron microscope (FE-SEM: LEO SUPRA 55, Carl Zeiss), a transmission electron microscope (TEM: JEM-2100F, JEOL), an X-ray diffractometer (M18XHF-SRA, Mac Science), and a fluorescence spectrometer (FluoroMate FS-2, Scinco) attached with a temperature-controlled heating holder operated in the temperature range of 25–250 °C. The lifetime for the samples was measured by using a Photon Technology International (PTI, USA) fluorimeter attached to a phosphorimeter with a Xe-flash lamp (25-watt power). Internal quantum efficiency was measured with the help of a fluoromax-4 spectrofluorometer (Horiba, Jobin Yvon) with an integrating sphere.

### pc-LEDs Fabrication

For preparing the pc-LEDs, a NUV LED chip with an excitation wavelength of 388 nm was employed. The NUV LED chip was first mounted on an LED frame and then slightly pressed to avoid gaps/air bubbles (formed after dispensing the phosphor mixed silicone encapsulant) between the chip and LED frame, which may damage the LED when a forward bias current is applied. The LED frame is then heated for few seconds for good attachment of LED and hardening of glue. Then, the translucent silicone epoxy was taken in 1:2 ratio and mixed well. Later, small amount of phosphors were added and mixed well with the help of a mixer. The final phosphor epoxy was desiccated to remove the air bubbles formed during the mixing and then taken into a dispenser tube. In the end, the phosphor paste was encapsulated onto the LED frame using a dispenser and then allowed to harden by heating (50 °C) for 90 min.

## Additional Information

**How to cite this article**: Bharat, L. K. *et al*. Rare-earth free self-luminescent Ca_2_KZn_2_(VO_4_)_3_ phosphors for intense white light-emitting diodes. *Sci. Rep.* **7**, 42348; doi: 10.1038/srep42348 (2017).

**Publisher's note:** Springer Nature remains neutral with regard to jurisdictional claims in published maps and institutional affiliations.

## Supplementary Material

Supplementary Information

## Figures and Tables

**Table 1:** Crystallographic and atomic parameters of the CKZV2 phosphor.

Crystallographic Data						
Crystal System				Cubic		
Space Group				Ia-3d (230)		
a (Å)				12.3903		
V (Å^3^)				1902.14		
R-Factors (%)						
χ^2^				3.72		
R_p_				5.0		
R_wp_				6.7		
**Atomic Parameters**						
**Atom**	**Wyckoff**	**Site**	**x/a (Å)**	**y/b (Å)**	**z/c (Å)**	**OCC**
Zn1	16 a	−3	0	0	0	0.1486
V1	24 d	−4	0.375	0	0.25	0.2565
Ca1	24 c	2.22	0.125	0	0.25	0.1757
O1	96 h	1	−0.0303	0.0496	0.166	0.9058
K1	24 c	2.22	0.125	0	0.25	0.0734
