# Supplementary material for: Rare-earth free self-luminescent Ca2KZn2(VO4)3 phosphors for intense white light-emitting diodes
Source: Sci Rep. 2017 Feb 9;7:42348. doi: 10.1038/srep42348 (PMC5299413; doi:10.1038/srep42348)
Supplement: Supplementary Information [file srep42348-s1.docx]

**Supporting Information**

**Rare-earth free self-luminescent Ca_2_KZn_2_(VO_4_)_3_ phosphors for intense white light-emitting diodes**

*L. Krishna Bharat^a^, Soo-Kun Jeon^b^, Kurugundla Gopi Krishna^a^ and Jae Su Yu^a*^*

^a^ Department of Electronic Engineering, Institute for Wearable Convergence Electronics, Kyung Hee University, Yongin-si, Gyeonggi-do 446-701, Republic of Korea

^b^ Semicon Light Co., Ltd., 49 Wongomae-ro 2beon-gil, Giheung-gu, Yongin-si, Gyeonggi-do 446-901, Republic of Korea

*Address correspondence to [jsyu@khu.ac.kr](mailto:jsyu@khu.ac.kr)

Tel: +82-31-201-3820; Fax: +82-31-206-2820

**Table S1:** Crystallographic and atomic parameters data for the CKZV1 and CKZV3 phosphors

|  | | | **Crystallographic Data** | | | | | | | |
| --- | --- | --- | --- | --- | --- | --- | --- | --- | --- | --- |
|  | | | **CKZV1** | | | | **CKZV2** | | | |
| **Crystal system** | | | Cubic | | | | Cubic | | | |
| **Space group** | | | Ia-3d (230) | | | | Ia-3d (230) | | | |
| **a (Å)** | | | 12.3855 | | | | 12.468 | | | |
| **V (Å^3^)** | | | 1899.93 | | | | 1938.28 | | | |
| **R-Factors (%)** | | |  | | | |  | | | |
| **χ^2^** | | | 4.36 | | | | 4.39 | | | |
| **R_p_** | | | 5.3 | | | | 6.7 | | | |
| **R_wp_** | | | 7.9 | | | | 8.0 | | | |
|  | | | **Atomic Parameters** | | | | | | | |
|  |  |  | **CKZV1** | | | | **CKZV2** | | | |
| **Atom** | **Wyckoff** | **Site** | **x/a (Å)** | **y/b (Å)** | **z/c (Å)** | **OCC** | **x/a (Å)** | **y/b (Å)** | **z/c (Å)** | **OCC** |
| Zn1 | 16 a | -3 | 0 | 0 | 0 | 0.1629 | 0 | 0 | 0 | 0.1672 |
| V1 | 24 d | -4 | 0.375 | 0 | 0.25 | 0.2487 | 0.375 | 0 | 0.25 | 0.2465 |
| Ca1 | 24 c | 2.22 | 0.125 | 0 | 0.25 | 0.1659 | 0.125 | 0 | 0.25 | 0.1642 |
| O1 | 96 h | 1 | -0.0308 | 0.0494 | 0.165 | 1.0000 | -0.0326 | 0.0516 | 0.164 | 1.0000 |
| K1 | 24 c | 2.22 | 0.125 | 0 | 0.25 | 0.0874 | 0.125 | 0 | 0.25 | 0.0896 |

**Table S2:** Comparison of internal quantum efficiencies of CKZV phosphor and some other vanadates

| **Phosphor** | **λ_Exci_ (nm)** | **CIE (x, y)** | **Q.E (%)** | **Reference** |
| --- | --- | --- | --- | --- |
| Ca_2_V_2_O_7_ | 350 | (0.494, 0.439) | 0.9 | [^1^](#_ENREF_1) |
| Sr_2_V_2_O_7_ | 350 | (0.393, 0.488) | 8 | [^1^](#_ENREF_1) |
| Ba_2_V_2_O_7_ | 350 | (0.227, 0.389) | 25.0 | [^1^](#_ENREF_1) |
| KVO_3_ | 347 | (0.362, 0.453) | 4.0 | [^2^](#_ENREF_2) |
| Mg_3_V_2_O_8_ | 340 | (0.449, 0.475) | 6.0 | [^3^](#_ENREF_3) |
| Ca_5_Zn_4_(VO_4_)_6_ | 320 | (0.425, 0.511) | 15.9 | [^4^](#_ENREF_4) |
| Ca_2_NaZn_2_V_3_O_12_ | 365 | (0.214, 0.338) | 11.4 | [^5^](#_ENREF_5) |
| Ca_2_KZn_2_V_3_O_12_ | 385 | (0.367, 0.476) | 19.2 | This work |


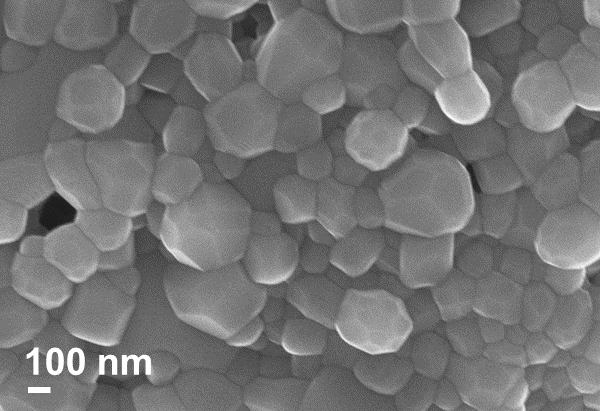


**Figure S1:** Low magnification SEM image of CKZV

**
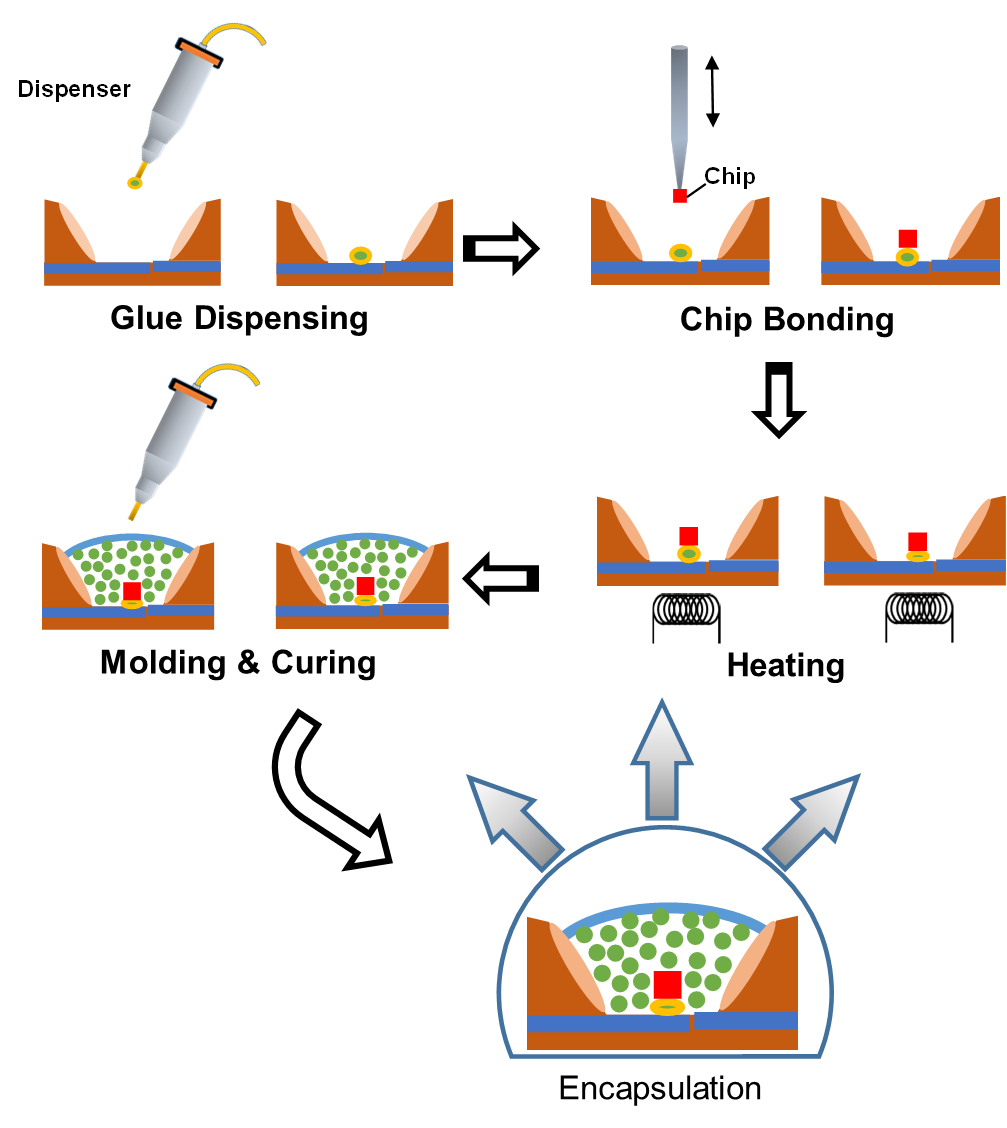
**

**Figure S2:** Schematic showing different steps for making a packaged WLED.

**
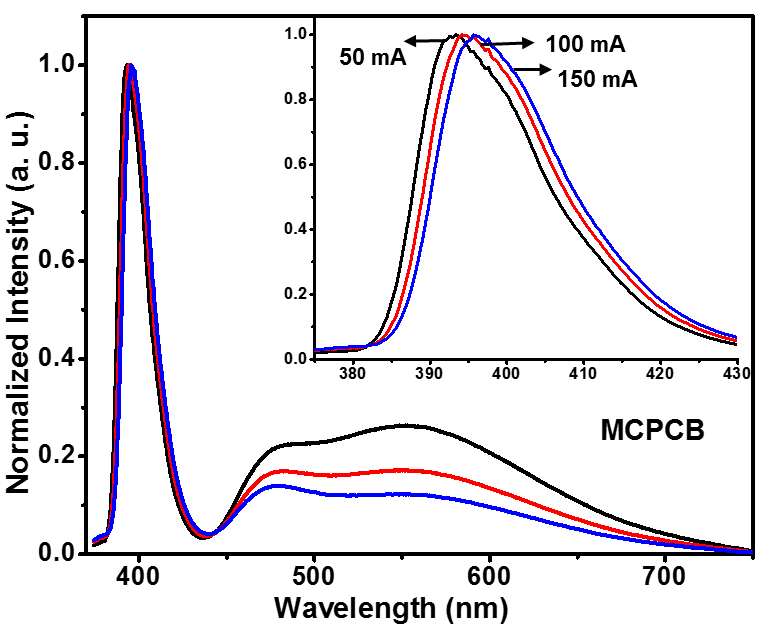
**

**Figure S3:** EL spectra of WLED with CKZV and blue phosphors mounted on MCPCB heat sink and measured at different operating currents.


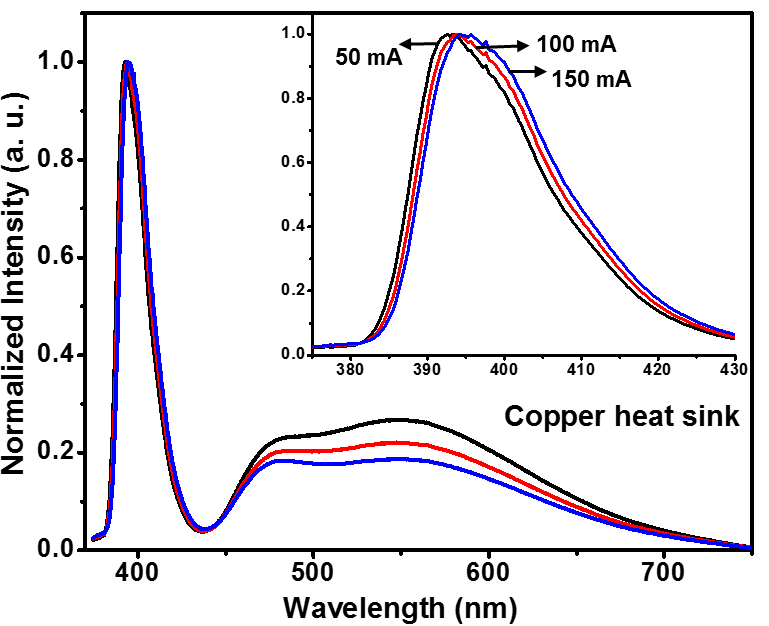


**Figure S4:** EL spectra of WLED with CKZV and blue phosphors mounted on copper heat sink and measured at different operating currents.

1 Nakajima, T., Isobe, M., Tsuchiya, T., Ueda, Y. & Manabe, T. Correlation between Luminescence Quantum Efficiency and Structural Properties of Vanadate Phosphors with Chained, Dimerized, and Isolated VO_4_ Tetrahedra. *J. Phys. Chem. C* **114**, 5160-5167, (2010).

2 Nakajima, T., Isobe, M., Tsuchiya, T., Ueda, Y. & Kumagai, T. Direct fabrication of metavanadate phosphor films on organic substrates for white-light-emitting devices. *Nat. Mater.* **7**, 735-740 (2008).

3 Nakajima, T., Isobe, M., Tsuchiya, T., Ueda, Y. & Kumagai, T. A revisit of photoluminescence property for vanadate oxides AVO_3_ (A:K, Rb and Cs) and M_3_V_2_O_8_ (M:Mg and Zn). *J. Lumin.* **129**, 1598-1601, (2009).

4 Huang, Y., Yu, Y. M., Tsuboi, T. & Seo, H. J. Novel yellow-emitting phosphors of Ca_5_M_4_(VO_4_)_6_ (M=Mg, Zn) with isolated VO_4_ tetrahedra. *Opt. Express* **20**, 4360-4368, (2012).

5 Chen, X., Xia, Z., Yi, M., Wu, X. & Xin, H. Rare-earth free self-activated and rare-earth activated Ca_2_NaZn_2_V_3_O_12_ vanadate phosphors and their color-tunable luminescence properties. *J. Phys. Chem. Solids* **74**, 1439-1443, (2013).
